# Effect on self‐awareness of autobiographical memory evoked by familiar olfactory stimulation in people with Alzheimer's disease in Bogotá, Colombia

**DOI:** 10.1002/alz70857_107000

**Published:** 2025-12-25

**Authors:** María Alejandra Castro Arbeláez, Daisy M Acosta, Eva María Arrollo‐Anlló, María Leonor Rengifo

**Affiliations:** ^1^ Universidad de Salamanca, Salamanca, Spain; ^2^ Instituto de Neurociencias de Castilla y León, Salamanca, Spain; ^3^ Asociación Dominicana de Alzheimer y Similares, Santo Domingo, Dominican Republic; ^4^ Universidad de la Sabana, Bogotà, Bogotá, Colombia; ^5^ Clínica de La Sabana, Chìa, Chía, Colombia; ^6^ Universidad Nacional Pedro Henriquez Urena (UNPHU), Santo Domingo, Santo Domingo, Dominican Republic; ^7^ Asociación Dominicana de Alzheimer y otras demencias, Santo Domingo, Santo Domingo, Dominican Republic; ^8^ Universidad de la Sabana, Chia, Cundinamarca, Colombia; ^9^ Clínica de la Sabana, Chía, Cundinamarca, Colombia

## Abstract

**Background:**

Smell is a sense that has been little explored in relation to Alzheimer's disease (AD) (El Haj, et al., 2019). Its study is essential because the (AD) continues to grow exponentially (Rocca et al., 2015; Iribarne et al., 2020). (Zurique‐Sánchez et al., 2019)., and there is no apparent cure within reach of everyone. Since non‐pharmacological approaches are involved, explorations in this sense could be accessible to a larger population, including vulnerable groups. Autobiographical memory is often diminished in Alzheimer's disease; however, semantic memory, which is linked to implicit memory and can be activated through olfactory stimuli, is less affected (El Haj, et al., 2019; Boccia, et. al, 2019; Arrollo‐Anlló et al., 2020). Therefore, olfactory stimulation could serve as a form of cognitive intervention that may positively impact self‐awareness. Previous studies have investigated the impact of sensory stimulations on Alzheimer's disease (AD) (El Haj, et. al, 2019, Glachet et. al, 2020), but few have done so through familiar olfactory stimulation, and none have considered the evocation of autobiographical memory and its impact on self‐awareness through a familiar scent in people with Alzheimer's in Latin America, specifically in Bogotá, Colombia.

**Method:**

An experimental procedure was applied using familiar olfactory stimulation and neuropsychological tests to assess autobiographical episodic and semantic memory in people with early and moderate‐stage Alzheimer's disease in the Bogotá region of Colombia in relation to self‐awareness. The experimental group included 25 people exposed to a familiar smell, and the placebo group included 25 people exposed to distilled water.

**Result:**

The preliminary results show changes in self‐perception in people with Alzheimer's disease. The final adjustments are expected to be completed by May 2025

**Conclusion:**

Considering the preliminary results, it could be anticipated that self‐awareness may improve in individuals with Alzheimer's disease following familiar olfactory stimulation with chocolate and lemon, taking into account autobiographical memory, in people with early and moderate stages of Alzheimer's in Bogotá, Colombia.

